# Checkpoint Inhibitors and Engineered Cells: New Weapons for Natural Killer Cell Arsenal Against Hematological Malignancies

**DOI:** 10.3390/cells9071578

**Published:** 2020-06-29

**Authors:** Massimo Giuliani, Alessandro Poggi

**Affiliations:** 1 Department of Oncology, Luxembourg Institute of Health, Luxembourg City L-1526, Luxembourg; 2 Molecular Oncology and Angiogenesis Unit, IRCCS AOU San Martino IST, 16132 Genoa, Italy; alessandro.poggi@hsanmartino.it

**Keywords:** NK cells, hematological malignancies, check-point inhibitors, CAR NK cells, antibodies, immunotherapy

## Abstract

Natural killer (NK) cells represent one of the first lines of defense against malignant cells. NK cell activation and recognition are regulated by a balance between activating and inhibitory receptors, whose specific ligands can be upregulated on tumor cells surface and tumor microenvironment (TME). Hematological malignancies set up an extensive network of suppressive factors with the purpose to induce NK cell dysfunction and impaired immune-surveillance ability. Over the years, several strategies have been developed to enhance NK cells-mediated anti-tumor killing, while other approaches have arisen to restore the NK cell recognition impaired by tumor cells and other cellular components of the TME. In this review, we summarize and discuss the strategies applied in hematological malignancies to block the immune check-points and trigger NK cells anti-tumor effects through engineered chimeric antigen receptors.

## 1. Tumor-Mediated NK Cell Exhaustion in Hematological Malignancies

NK cells represent one of the first lines of defense against malignant cells. Their immune-surveillance ability is mediated by the expression of activating receptors such as the natural killer group (NKG)2D, DNAX accessory molecule (DNAM)-1, and the natural cytotoxic receptors (NCRs) such as natural killer protein (NKp)30, NKp44, and NKp46 [1,2,3,4,5]. NK cell recognition is also dependent by an array of inhibitory receptors, such as killer inhibitory receptors (KIRs) and NKG2A molecule [6]. Once the target is acquired in the viewfinder, NK cells secrete cytotoxic granules (as granzymes and perforin) and cytokines (as tumor necrosis factor (TNF)-α and interferon (IFN)-γ), which lead to the killing of the target. 

**Figure 1:**
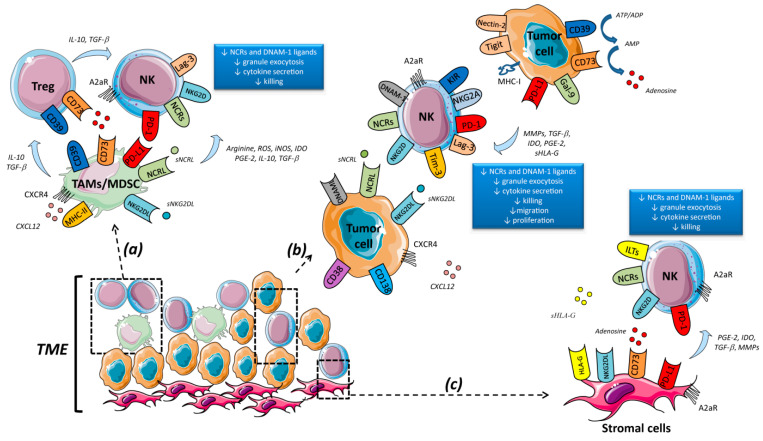
Strategies used by the tumor microenvironment (TME) to impair natural killer (NK) cell immuno-surveillance in hematological malignancies. (**a**) Tumor cells secrete several chemokines as CXCL12 to recruit suppressive cells such as myeloid derived suppressors cells (MDSCs) and tumor–associated macrophages (TAMs). These cells inhibit NK cell functions by secreting soluble factors such as interleukin (IL)-10, transforming growth factor (TGF)-β, reactive oxygen species (ROS), arginine and nitric oxide synthase (NOS), or through the expression of inhibitory receptors as programmed death-ligand (PD-L)1 or release of ligands for NK activating receptors. In addition, MDSCs and TAMs can recruit other suppressive cells like regulatory T cells (Tregs), which indirectly contribute to induce an exhausted and dysfunctional profile in NK cells. (**b**) Tumor cells secrete immunosuppressive molecules whose impair NK cell proliferation, activation and cytotoxicity, such as TGF-β, prostaglandin (PG)E-2, indoleamine 2,3-dioxygenase (IDO) and soluble human leukocyte antigen (HLA)-G. A mechanism used by hematological malignancies to avoid NK cell-mediated recognition is the expression of inhibitory receptors as PD-L1 Also, tumor cells can secrete natural killer group (NKG)2DLs, which impair the interaction between tumor and NK cells affecting the positive signal induced by NKG2D. (**c**) Mesenchymal stromal cells (MSC) decrease granule exocytosis, cytokines secretion and cytotoxicity of NK cells through the secretion of soluble factors as PGE-2, TGF- β and soluble HLA-G and through the expression of PD-L1 and HLA-G.

Several molecular mechanisms have been developed in hematological malignancies to allow tumor cells to elude and escape from NK cell-mediated recognition. Some of these mechanisms are represented by the inhibition of tumor antigen presentation, expression of immune checkpoint ligands as programmed death ligand-1 (PD-L1), secretion of suppressive factors like interleukin (IL)-10, soluble human leukocyte antigen (HLA)-G, transforming growth factor (TGF)-β, indoleamine 2,3-dioxygenase (IDO), recruitment and polarization of immunosuppressive cells as macrophages, regulatory T cells (Tregs), myeloid derived suppressor cells (MDSC), and mesenchymal stromal cells (MSC). Altogether these cells, with the tumor cells themselves, are present inthe tumor microenvironment (TME) [7,8,9,10,11] (Figure 1). Another major molecular mechanism used by tumor cells to impair NK cell recognition and activation is based on the expression of inhibitory (as major histocompatibility complex (MHC) class I molecules) and the release in a soluble form of ligands (such as MHC class I polypeptide–related sequence (MIC) A/B and UL16 binding protein (ULBP1-6) for NK cell-activating receptors [12,13]. The TME is insomuch efficient that NK cells isolated from patients with hematological malignancies display multiple abnormalities. In particular, NK cells isolated from chronic myeloid leukemia (CML), acute lymphocytic leukemia (ALL), myelodysplastic syndromes (MDS), and chronic lymphocytic leukemia (CLL) patients suffer a decreased cell number, activating receptors expression, and cytokines secretion [14,15,16,17,18,19]. Similar tumor-mediated impairment of NK cell functions has been also described in acute myeloid leukemia (AML) and multiple myeloma (MM) patients [15,16,17,20,21,22,23,24]. This is associated with an impaired polarization of cytolytic granules toward the immunological synapse against tumor cells [16,23,25,26] and increased expression of inhibitory receptors like NKG2A, programmed death receptor (PD)-1 and KIRs [27,28,29,30]. Interestingly, it has been shown in CLL patients that NK cells losing NKp30 on their surface acquire the inhibitory receptor T-cell immunoglobulin and mucin domain (TIM)-3, which was correlated with poor prognostic factors [31]. 

## 2. Current Advanced Therapy in Hematological Malignancies

Once effectors cells infiltrate the tumor site, they have to fight both tumor cells and the other components of the TME. To that end, the purpose of recent therapeutic strategies is to improve NK cell survival, proliferation, activation, and cytotoxic functions in a hostile and immune-suppressive environment. Over the years, several approaches have been developed in hematological malignancies, like monoclonal antibodies (mAbs) and engineered NK cells.

### 2.1. Monoclonal Antibodies (mAbs)

**Figure 2:**
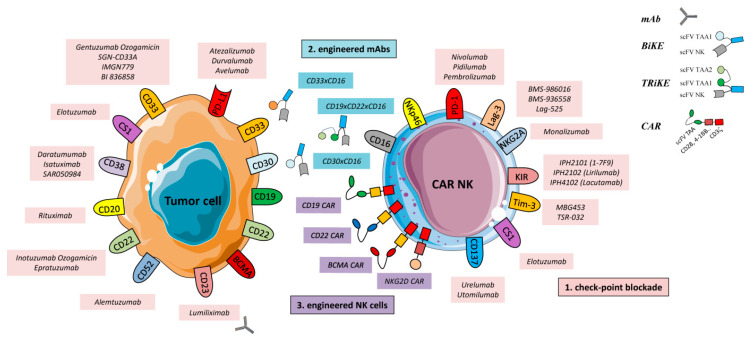
Overview of emerging strategies to boost or restore NK cell-based anti-tumor response in hematological malignancies. (**1**) Checkpoint blockade. MAb-based therapy is an approach aimed to block the triggering of inhibitory receptors (as PD-1, NKG2A, and KIRs) expressed on NK cells and avoid tumor escape. Importantly, checkpoint inhibition can be also used to impair tumor cell functions through specific mAbs as Durvalumab (anti-PD-L1), Daratumumab (anti-CD38), or Elotuzumab (anti-CS1). (**2**) Engineered mAbs. BiKEs and TRiKEs bind to activating receptors (e.g., CD16) expressed on NK cells and several antigens (e.g., CD19, CD22, CD33, CD38, and CD123) expressed on tumor cells. Engineered mAbs facilitate the formation of an immunological synapse (IS) and improve ADCC activity by redirecting NK cells to tumor cells.(**3**) CAR NK cells are genetically modified to recognize specific antigens expressed on tumor cells. The consequence of the CAR activation is the formation of a strong IS, followed by the release of cytotoxic granules as perforin and granzymes and eventually the target cell killing. Abbreviations used: ADCC, antibody-dependent cell-mediated cytotoxicity; mAbs, monoclonal antibodies; BiKEs, bi-specific killing cell engagers; TRiKEs, tri-specific killer cell engagers; CARs, chimeric antigen receptors.

In hematological malignancies, tumor cells and tumor-associated cells express activating and inhibitory receptors thataffect anti-tumor response. The monoclonal antibody-based therapy is an approach aimed to block the triggering of these receptors, with the purpose to target the entire TME and restore NK cell functions [32,33,34,35,36] (Figure 2). Herein, a list of mAbs and target molecules are analyzed in this context.

Rituximab and other more recent anti-CD20 mAbs, such as Ocaratuzumab and Ublituximab, have been shown to improve the clinical prognosis in B-cell hematologic malignancies, such as diffuse large B-cell lymphoma (DLBCL), CLL, and follicular lymphoma (FL) [37,38,39]. Interestingly, it has been shown that NK cells isolated from CLL, lymphomas, and Waldenström Macroglobulinemia (WM) patients treated with Rituximab, Ocaratuzumab, or Ublituximab displayed an increased antibody-dependent cellular cytotoxicity (ADCC) and degranulation function [40,41,42,43,44]. Moreover, the combined blockade of KIRs and CD20 with specific mAbs enhanced NK cell cytotoxicity against lymphoma cells in vitro and in murine lymphoma models [41]. By contrast, it has been also observed that Rituximab and Ofatumumab promoted the release of reactive oxygen species from monocytes, which impaired the NK cell-mediated ADCC against CLL cells [45].

**Elotuzumab** is a mAb interacting with the glycoprotein signaling lymphocytic activation molecule F7 (SLAM-F7, also named CS1 or CD319) expressed on malignant plasma cells.Notably, SLAM-F7 is also expressed on NK cells, and it has been reported that Elotuzumab enhances NK cell-mediated anti-myeloma activity by directly activating NK cells, inducing ADCC and disrupting the stromal/MM cell interaction [46,47,48,49,50,51]. In addition, Elotuzumab has been shown to improve the overall response rate in patients with refractory/relapsed (r/r) MM [52,53,54], and its efficacy can be enhanced by simultaneous treatment with drugs as Carfilzomib and Panobinostat [46,55], Bortezomib [56,57], or immunomodulatory drugs (IMiDs) [46,53,58,59,60]. Importantly, Elotuzumab action might be also enhanced when used combined with other mAbs. A phase I open-label study of the safety and tolerability of Elotuzumab (BMS-901608) administered in combination with either Lirilumab (BMS-986015, anti-KIR) or Urelumab (BMS-663513, anti-CD137) (NCT02252263) is under investigation in MM patients. In addition, three distinct clinical trials are investigating the efficacy between Elotuzumab and Nivolumab [an anti-Programmed Death-1 (PD-1) mAb] in r/r MM patients [NCT02726581 (CheckMate-602, phase III)], (NCT02612779, phase II), and (NCT03227432, phase II) (ClinicalTrials.gov) (Table 1).

CD137/4-1BB is a co-stimulatory molecule expressed on T and NK cells, whose triggering efficiently improved CTL-mediated tumor killing [61,62,63]. Recent clinical trials have demonstrated the promising effect of anti-CD137 agonistic mAbs as Urelumab (BMS-663513) and Utomilumab in hematological malignancies, alone or combined to other mAbs as Lirilumab, Elotuzumab, Rituximab, or Nivolumab [34,64,65,66,67].

- Adenosine, CD39, and CD73

The adenosine, generated by the ectonucleotidases CD39 and CD73, has been recently proposed as a novel target since it plays a key role in the inhibition of anti-tumor response through the activation of adenosine receptor (A2AR) expressed on effectors cells [68,69]. Interestingly, CD73 and CD39 are not only expressed on tumor cells, but also other components of the TME as tumor-associated macrophages (TAMs), MDSC, Tregs, and MSC [70,71]. Noteworthy, the adenosine secreted by the TME impairs NK cell proliferation, activation, and killing abilities [72,73,74] (Figure 1). CD73 is usually not expressed in healthy NK cells. Nonetheless, it has been reported that CD73 expression is up-regulated in tumor-infiltrating NK cells [75]. Further, these CD73^+^ NK cells also express other inhibitory checkpoints and they can suppress CD4^+^T-cell proliferation and IFN-γ production, thus promoting tumor growth. Notably, CD73 and CD39 are not only expressed on solid tumors but also in hematological malignancies [69,76,77,78,79]. More interestingly, it has been reported that the expression of CD39 on MSC increases significantly after co-culture with activated lymphocytes [80]. Along with this, Chatterjee et al. found that NK cells co-cultured with MSC displayed a significant up-regulation of CD73 expression, suggesting the possibility that CD73^+^ NK cells could convert AMP into adenosine upon exposure to MSC, hence maintaining the immune-suppressive milieu promoted by other cellular components of the TME [81]. Thus, MSC display a strong talent to suppress the immune response by converting NK cells in inhibitory partners; altogether these results highlight the importance to consider MSC a suitable target in immunotherapy [9,10,11]. In the past decade, several pharmacological inhibitors and specific mAbs have been evaluated in pre-clinical studies. Although the blockade of CD73 and/or A2AR signaling has been shown to restore effectors cell functions and inhibits tumor growth in solid tumors [70,71,82,83], there are no ongoing studies in hematological malignancies.

- Other Molecular Targets for NK Cell Immunotherapy

Other promising strategies under investigation include the targeting of the **CD38**, which is highly expressed on both normal and malignant plasma cells. Indeed, it has been shown that the anti-CD38 mAb Daratumumab enhances effectors cell-mediated lysis, degranulation, and ADCC against CD38^+^ tumor cells [84,85,86,87] improving the overall response rate in MM patients [88,89,90,91]. Interestingly, the efficacy of Daratumumab can be enhanced when combined with drugs or other mAbs [85,90,92]. Other anti-CD38 mAbs under investigation in hematological malignancies are Isatuximab and MOR-202 [93,94,95]. A detailed summary of ongoing and completed clinical trials using anti-CD38 mAbs are listed recently [85,90,91] (Table 1). Other promising targets of mAbs are **CD52** (Alemtuzumab, in B-ALL precursors), **CD23** (Lumiliximab, in CLL), **CD22** (Inotuzumab Ozogamicin and Epratuzumab, in precursors and mature B-ALL), and **CD33** (Gemtuzumab ozogamicin, in AML) [39,96,97]. Remarkably, it has been recently shown that an Fc-engineered CD33 mAb, BI-836858, promotes NK cell-mediated ADCC with in vitro activity against both AML cell lines and primary AML blasts [98]. Several findings suggest that **CD47** could be another target for cancer immunotherapy in hematological malignancies. CD47 is principally expressed on myeloid cells and it is exploited by tumor cells to evade immune response [99,100,101]. Drugs targeting the CD47 signaling are currently evaluated in clinical studies and are represented by humanized antibodies including Hu5F9-G4 [(in AML, MDS and r/r B-cell Non-Hodgkin Lymphoma (NHL)] and CC-90002 (in AML, MDS and CD20^+^ NHL patients), respectively.

### 2.2. Checkpoint Inhibitors

- Anti-KIRs and Anti-NKG2A mAbs

As postulated by the “missing-self hypothesis,” the absence (or low expression) of MHC class I molecules on tumor cells trigger NK cells and leads to the tumor cell killing [2]. By contrast, the NK cell cytotoxicity is impaired when the tumor target expresses appropriate MHC class I alleles interacting with the KIRs expressed on NK cells. To improve NK cell functions, several strategies to block these KIRs have been developed. The blockade of KIRs with IPH2101 (formerly 1-7F9), an anti-pan-KIR antibody which interacts with KIR2DL1, KIR2DL2, and KIR2DL3 expressed on NK cells, strongly increases NK cell-mediated killing of tumor cells in AML, lymphoma, and MM patients [41,102,103,104,105,106,107]. IPH2101 efficacy has been tested in a phase I study in MM combined with the IMiD Lenalidomide (NCT01217203) [103]. Other anti-KIR mAbs are represented by Lirilumab (IPH2102/BMS-986015) and IPH4102, which interacts with KIR2DL1, KIR2DL2 and KIR2DL3, and KIR3DL2, respectively. Interestingly, IPH2102 has been shown to increase NK cell lysis against lymphoma cells [41] and synergistically acts with Lenalidomide to improve Daratumumab-treated MM cells lysis mediated by NK cells [86]. The effect of Lirilumab has been investigated in several hematological malignancies such as in a phase Ib/II study of relapsed AML in association with 5-Azacytidine (NCT02399917), for r/r or high-risk untreated CLL, treated with Rituximab (NCT02481297) in MM patients with Elotuzumab (BMS-901608) or Urelumab (BMS-663513) (NCT02252263, phase I), in MM and r/r lymphoma patients with Nivolumab (NCT01592370, phase II) [108], in MDS patients with Nivolumab and 5-Azacitidine (NCT02599649, phase II) and elderly AML patients in first complete remission as maintenance treatment (NCT01687387, phase II) [35,109]. The NKG2A ligand HLA-E is strongly expressed on malignant plasma cells [110]. Monalizumab (formerly IPH2201) blocks the inhibitory signaling induced by NKG2A/CD94 expressed on NK cells, restoring the anti-tumor response mediated by NK cells in hematological malignancies [30,61,111,112,113]. Also, Monalizumab is currently tested in a phase Ib/IIa study combined with Ibrutinib in patients with r/r CLL patients (NCT02557516) and a phase I study combined with Durvalumab (MEDI4736, an anti-PD-L1 antibody) in solid tumors (NCT02671435) (Table 1). Finally, the simultaneous blockade of NKG2A, the leukocyte-associated Ig-like receptor-1 (LAIR-1), and KIRs have been shown to strongly increase the NK cell-mediated killing of AML and ALL blasts [114].

- HLA-G

HLA-G binds to immunoglobulin-like transcripts (ILT)-2, ILT-4 and KIR2DL4 [115]. ILTs are expressed by most of the immune cells, including NK cells. ILT-2/HLA-G interaction impairs several functions on NK cells, such as cytokine secretion, chemotaxis, and the immunological synapse formation between NK cells and their target [115]. Of note, HLA-G belongs to the immunosuppressive factors secreted by the TME components in hematological malignancies, which contributes to the immune evasion of tumor cells [7,8,9,10,11,115,116]. Although it has been reported that Lenalidomide decreases the expression of ILT-2 on CLL cells, thus promoting NK cell proliferation and activation [117], today, there are no clinical studies evaluating the possible inhibition of HLA-G (or its receptors) in NK cell-based immunotherapy in hematological malignancies.

- Lymphocyte-activation gene-3 (LAG-3), TIM-3, PD-1, and T cell immunoglobulin and ITIM domain (TIGIT)

Associated to the increased expression of KIRs and NKG2A, exhausted effectors cells can exhibit elevated levels of inhibitory receptors such as lymphocyte activation gene-3 (LAG-3), TIM-3, and PD-1. These inhibitory receptors are currently under clinical investigation as potential therapeutic targets.

LAG-3 is expressed on B, T, and NK cells and binds to MHC class II molecules and L-SECtin bearing tumor cells [34,35,118]. However, LAG-3 is also expressed on PD-1^+^ tumor-infiltrating effectors cells (TILs) found in both pre-clinical models and patients, where they promote tumor escape. These PD-1^+^LAG-3^+^TILs exhibited an exhausted profile, characterized by reduced cytokines and cytotoxic granules secretion [119,120,121,122]. LAG-3^high^ expression is associated with poor outcome in several hematological malignancies; ongoing clinical studies are evaluating the effect of anti-LAG-3 mAbs administered alone or in combination with other mAbs as Nivolumab [34,35,118,123].

TIM-3 is expressed by Tregs, DC, and T cells. TIM-3 interacts with Galectin-9, carcinoembryonic antigen-related cell adhesion molecule (CEACAM)-1, high-mobility group box (HMGB)-1, and phosphatidyl serine; these TIM-3 ligands are expressed not only by other immune cells but also by B-cell malignancies [34,35,118,119,124]. Similarly to LAG-3, the expression levels of TIM-3 on TILs correlates with cell dysfunction or exhaustion and poor prognostic factors, especially when TIM-3 is associated with other inhibitory receptors as PD-1 [35,125]. Interestingly, mature CD56^dim^CD16^+^ NK cells express TIM-3, and its expression can be induced upon activation. Intriguingly, compared with T cells, TIM-3^high^ NK cells are fully activated and able to secrete cytokines and kill their targets. Thus, TIM-3 blockade may suppress NK cell-mediated cytotoxicity [124,126,127,128]. Notably, in hematologic malignancies TIM-3 (or its specific ligand Galectin-9) blockade restores immune response in AML, follicular lymphoma (FL), and lymphoma [124,126,129]. Interestingly, it has been recently shown that TIM-3 is also expressed on MDS blasts, and this expression is further enhanced in the presence of the cell culture supernatant of human stromal cell lines [130]. Based on these findings, the anti-TIM-3 mAb MBG453 is currently evaluated in a phase I clinical trial for r/r AML and high-risk MDS, combined with Decitabine, a nucleic acid synthesis inhibitor (NCT03066648).

PD-1 (or CD279) is expressed on both tumor and activated immune cells, and when it interacts with its specific ligands, it induces effectors cells exhaustion with consequent tumor progression [36,131,132]. This mechanism has been observed in most of hematological malignancies, PD-1 being expressed on AML [133,134], MM [27,135,136,137], NHL [138,139], DLBCL [140,141], and CLL cells [142]. In addition, TILs exhibiting PD-1 have been observed in FL [143,144,145] and NHL [146]. Of note, PD-1 is expressed on CD56^dim^/NKG2A^-^/KIR^+^/CD57^+^ NK cells, which correspond to a terminally differentiated and exhausted status, characterized by a decreased proliferation, cytokine secretion, and degranulation [147,148]. Interestingly, it has been recently observed in FL the presence of two different T-cell subsets displaying opposite localization and functions, based on the expression of PD-1 [146]. Whereas PD-1^high^T cells, which predominantly reside in the lymph node follicles, are TIM-3^-^, secrete IL-21 and support B-cell growth, PD-1^low^T cells (mainly located in an inter-follicular pattern), have an exhausted phenotype, express TIM-3, and predict a poor outcome in FL patients. Noteworthy, recent evidence supports the fact that expression of PD-1 on lymphocytes in myeloma patients is lowered during Lenalidomide maintenance [149]. Interestingly, in several studies anti-PD-1 mAbs were combined with drugs as IMiDs [27,55,150,151,152], 5-azacytidine [153,154], Rituximab [155], or others checkpoint inhibitors as anti-LAG-3 [121] or anti-CTLA-4 antibodies [154,156].

The ligands for PD-1 are PD-L1 (CD274 or B7-H1) and PD-L2 (CD273 or B7-DC). PD-L1 expression can be modulated by epigenetic and post-transcriptional modifications, Toll-like receptor-mediated signaling, and the surrounding TME [27,137,157,158]. Paradoxically, PD-L1 expression is also up-regulated by the IFN-γ secreted by anti-tumor effectors cells; this may amplify PD-L1-mediated immunosuppressive effects [159]. Recent evidence has also shown that PD-L1 is expressed in different hematological malignancies [140,141,160,161,162,163]. There are several groups of mAbs used to disrupt the PD-1/PD-L1 axis, aimed to restore anti-tumor response. Whereas Nivolumab (MDX1106, BMS-936558), Pembrolizumab (MK-3475), and Pidilizumab (CT-011) block the PD-1-induced inhibitory signaling, BMS935559 (MDX-1105), MPDL3280A, and MEDI4736 (Atezolizumab, Durvalumab and Avelumab, respectively) affect the inhibitory signaling induced by PD-L1. Remarkably, it has been shown that the anti-PD-1/PD-L1 blockade restored NK cell cytotoxicity against MM cells [27,136,164,165,166,167]. Interestingly, it has been recently reported that combining a selective HDAC6 inhibitor (ACY-241) with an anti-PD-L1 mAb triggers the effector cell-mediated MM cell killing, supporting their utilization in the clinical studies aimed to restore immune response [137,157,168]. Based on these findings, several clinical studies are evaluating the therapeutic efficacy of both anti-PD-1 and anti-PD-L1 mAbs in most of hematological malignancies [35,109,139,150,169,170]. Another ligand of PD-1 is PD-L2. This molecule is also expressed in hematological cancers, where it participates inthe immune-tolerance [171,172,173,174,175]. Although further investigations are needed, altogether this evidence suggests that a possible strategy to induce the full restoration of the anti-tumor response could be the use of anti-PD-1/PD-L1 and anti-PD-L2 antibodies.

TIGIT is expressed on both activated T and NK cells and interacts with two specific DNAM-1 (CD226) ligands, CD155 (PVR) and CD112 (nectin-2), which are expressed on both immune and tumor cells [66,119,176,177]. Importantly, TIGIT binds CD155 with stronger affinity than DNAM-1; TIGIT interacting with both CD155 and CD112 promotes the decrease of IFN-γ production and NK cell-mediated cytotoxicity [178,179,180,181]. Importantly, both TIGIT and CD155 must form homodimers in *cis* to interact as heterotetramers in *trans*. This molecular mechanism is also used by DNAM-1, but it is inhibited by TIGIT, allowing an impaired anti-tumor response mediated by effectors cells (reviewed in [182,183]). Interestingly, TIGIT expressed on tumor-infiltrating effector cells synergizes with other co-inhibitory molecules to dampen the immune response and promote effector cells dysfunction [184,185], so that the co-blockade of TIGIT/PD-1/TIM-3 restored exhausted CD8^+^ T cells and induced complete tumor rejection [116,176,186,187]. Noteworthy, TIGIT ligands are also expressed in hematological malignancies, where they induce T-cell dysfunction associated with a poor clinical prognosis [188,189,190]. The nuisance is that TIGIT^+^PD-1^+^TIM-3^+^ [190] or TIGIT^+^PD-1^+^DNAM-1^-^ [189] T cells exhibit strongly impaired cytokines secretion ability, which can be restored by blocking TIGIT, PD-1, and TIM-3 altogether [190]. Furthermore, the expression of DNAM-1 ligands on malignant plasma cells triggers human NK cell-mediated cytotoxicity against MM cells [20,187]. Noteworthy, TIGIT ligands CD112 and CD155 are not only highly expressed on AML cells, but the blockade of the TIGIT/CD112/CD155 axis augments T cell-mediated lysis of AML cells and enhances the cytotoxic effects of the CD33/CD3 bi-specific T cell engager (BiTE)^®^ antibody construct AMG-330 [191,192]. Although evaluated only in solid tumors, this evidence indicates that TIGIT could represent a potentially promising target also for the treatment of hematological malignancies [34,116]. 

Another receptor expressed on NK cells showing great interest is the T-cell activation increased late expression (TACTILE) molecule or CD96. TACTILE is constitutively expressed on resting NK cells; it can interact with CD155 and it appears to inhibit NK cell-mediated IFN-γ production in mice, while it may enhance NK cell-mediated cytotoxicity in humans. These contrasting effects make unclear the clinical significance of TACTILE targeting [119,177,180,187]. Interestingly, DNAM-1 and TACTILE induce two opposite signals when they interact with CD155. Whereas the complex DNAM-1/CD155 activates NK cells, the interaction TACTILE/CD155 leads to a strong reduction of cytotoxicity, granule polarization, and cytokine secretion in NK cells [116,180,184,185]. Moreover, TACTILE can be expressed by malignant plasma cells in AML, T-cell acute lymphoblastic leukemia (T-ALL), and myelodysplastic syndromes [184]. Despite a possible interest as a potential target for the treatment of hematological malignancies, in humans, the role of TACTILE in NK cells functions is not completely understood, because of the presence of both activating and inhibitory motifs.

- Other molecular Targets for NK Cell-Mediated Immunotherapy

An inhibitory receptor expressed on NK cells under investigation is sialic acid-binding Ig-like lectin-7 (Siglec-7) which dampens NK cell surveillance and lead to tumor cells escape [7,193,194,195]. Interestingly, Siglec-7^+^ NK cells strongly express CD16, DNAM-1, NKp30, and NKp46, and exhibit a strong CD107a degranulation and IFN-γ production [195]. Of note, several Siglec-7 ligands have been detected on NK cells including the ganglioside disialosyl globopentaosylceramide (DSGb5) [196] and the ganglioside GD3 [197]; the interaction of Siglec-7 with these two gangliosides can modulate NK cell-mediated cytotoxicity against kidney carcinoma cells and P815 mouse mastocytoma cell line. Importantly, Siglec ligands are expressed at tumor cell surface and they seem to play an important role in the tumor escape from NK cell-mediated immunosurveillance [193]. An exhaustive summary of Siglec ligands has been reported by [193,198]. In hematological malignancies, Siglec-7 ligands have been observed in CML, CLL, AML [199], and MM [193,194] cells. 

Another attractive target for cancer immunotherapy is B7-H3 (CD276); this molecule plays a key role in the inhibition of T-cell function [34,200,201,202,203,204] and it is highly expressed on a wide range of human solid cancers; Its expression often correlates with both negative prognosis and poor clinical outcome of patients [202,203]. The B7-H3-mediated functions remain poorly investigated in hematological malignancies. To our knowledge, B7-H3 has been reported expressed only by AML cella [205,206] and mantle cell lymphomas (MCL) [207]. Interestingly, a bi-specific antibody CD3/B7-H3 (B7-H3Bi-Ab) has been reported to enhance the ability of T cells to secrete cytotoxic granules and cytokines, associated with the killing of hematological tumor cells [208]. Another inhibitory receptor expressed on NK cells is CD161 (NKR-P1A). CD161 can bind to C-type lectin-like transcript-1 (LLT-1) expressed by several hematological malignancies, including Burkitt lymphoma, FL, and DLBCL [209,210]. It is of note that the CD161/LLT1 interaction in NK cells impairs cytokines secretion and cytotoxic activity, thus decreasing tumor susceptibility to NK cells [209,210,211]. The negative role of LLT-1 on NK cell functions is confirmed by the fact that the blockade of CD161/LLT-1 axis increases the NK cell-mediated secretion of IFN-γ and the killing of tumor cells [210,211].

Finally, Polatuzumab vedotin is amAb recognizing the B-cell receptor component CD79b. This antibody is currently under investigation in hematological malignancies [212]. In r/r DLBCL patients, it has been used combined with bendamustine (an alkylant agent) and Obinutuzumab (an anti-CD20 mAb) (NCT02257567, phase Ib/II) [213], or in combination with Rituximab or Obinutuzumab and Cyclophosphamide, Doxorubicin, and Prednisone (NCT01992653, phase I/II) [214]. Also, in r/r NHL patients, Polatuzumab has been used in combination with Rituximab (NCT01691898, phase II) [215] (Table 1).

### 2.3. Engineered mAbs 

#### 2.3.1. Bi-specific T cell Engagers, Bi-Specific Killer Engagers, and Tri-Specific Killer Engagers (BITEs, BIKEs, and TRIKEs) 

As discussed above, mAb-based therapy represents an important tool to promote an efficient anti-tumor immune response. At present, this therapeutic tool has been “further improved” with the development of bi-specific antibodies. These antibodies are engineered proteins recognizing simultaneously two different antigens: one target antigen is expressed on tumor cells and the second one is an activating receptor expressed on immune effector cells. Thus, bi-specific T-cell engagers (BiTEs) represent a promising approach, since effector cells stimulated with BiTEs display an increased expression of CD69 and CD25, with consequent effector cell proliferation, cytokine and cytotoxic granules secretion, leading to a strong anti-tumor response [216,217,218,219,220,221]. Noteworthy, several BiTEs are currently investigated in clinical trials in hematological malignancies for their safety and efficacy. Based on the BiTE’s philosophy, Bi-specific killing cell engagers (BiKEs) have been developed to improve NK cell functions by facilitating their interaction with the target, principally through the CD16 activation [222,223] (Figure 2). In vitro studies have demonstrated that the BiKE CD16 × CD33 increases NK cell cytotoxicity and cytokine production in AML [224] and MDS, respectively [224,225]. Other BiKEs are represented by CD16 × CD19 and CD16 × CD133, whose engagement promote NK cell activation against CD19^+^ and CD133^+^ tumor cells, respectively [61,226]. Based on results showing that the bi-specific antibody CD30/CD16A (AFM13) can enhance NK cell cytotoxicity against CD30^+^ HL cells, this BiKE is currently under evaluation in a clinical study (NCT01221571, phase I) to assess its safety in HL patients [225,227,228,229,230]. Other clinical studies have been performed, or are ongoing analyzing, the effect of CD19/CD16 [15,40,224,226,229], CD123/CD3 [15], and CD20 × CD16 [231] in hematological malignancies [220,232,233,234,235,236].

Recently, several tri-specific killer cell engagers (TriKEs) have been also developed to boost NK cell functions [222,223,224,225,226,227,228,229,230,231,232,233,234].TriKEs work similarly to BiKEs, indeed they bind to an activating receptor expressed on NK cells (e.g., CD16) and to two different antigens expressed on tumor cells; this can lead to the generation of a very strong immunological synapse between tumor and effector cells. For example, the CD16 × CD19 × CD22 or CD16 × CD33 × IL-15 TriKEs trigger NK cell activation, ADCC and cytokine secretion leading to the release of lytic granules against B-cell leukemia and AML cells [15,233,237,238]. Interestingly, the TriKE CD16 × CD33 × IL-15, in which one of the antibodies has been substituted with an immunostimulating cytokine such as IL-15, has been also reported to stimulate NK-cell function to overcome immune suppression mediated by MDSCs in MDS [239]. Also, Glorius et al. have demonstrated that “tri-body” engagers CD20 × CD20 × CD16 efficiently trigger effectors cell-mediated lysis of malignant B cells [231]. Remarkably, it has been recently reported by Gauthier and colleagues the possibility to generate tri-functional NK cell engagers (NKCEs), which can target NKp46 and CD16 on NK cells and a tumor antigen on cancer cells [210,240]. Noteworthy, in the attempt to improve the anti-leukemic specificity of activated NK cells, others have investigated ULBP2 (an NKG2D ligand) × CD19 × CD33 engagers [triple-bodies (TBs)] [241].

#### 2.3.2. Dual-Affinity Re-Targeting T cells (DARTs) 

Although today this strategy is not currently investigated in NK cells, the dual-affinity re-targeting T cells (DARTs^®^) merit to be mentioned. Indeed, it has emerged as a promising tool in the treatment of hematological malignancies. Similarly to BiTEs, DARTs trigger CD3 on T cells and a specific tumor-associated antigen (TAA) on malignant cells (e.g., CD19). Stimulated T cells are then able to kill tumor cells in vitroand to suppress tumor growth and induce tumor regression [96,242,243].

## 3. Engineered Effector Cells 

### 3.1. Chimeric Antigen Receptor (CAR) NK Cells

In the past decade, besides the generation of several kinds of mAbs used in immunotherapy, also some engineered anti-tumor immune cells have been developed. Indeed, the chimeric antigen receptors (CARs), have been transduced in effector T lymphocytes of tumor bearing patients to improve their anti-tumor response. These CARs were composed of an extracellular domain able to recognize the tumor and an intracellular portion that delivers an activating signal to T lymphocytes. Thus, CAR T cells are ready-to-kill effector cells, equipped to migrate at the tumor site, circumvent TME traps, and then attack tumor cells [140,244,245,246,247,248,249]. While the 1st generation of CARs contained only one intracellular co-stimulatory domain (e.g., CD3ζ), the recent generations contain several co-stimulatory domains (e.g., CD3ζ and CD28 and CD137/4-1BB), which enhance long-term T-cell activation and are used in most recent clinical trials. To interact with a specific tumor-associated antigen(TAA), such as CD19 on neoplastic B cells, CAR-T cells express an extracellular domain with a single-chain fragment variable (scFv) derived from an anti-CD19 antibody. This scFv promotes the interaction with CD19 on tumor B cells and through the intracellular domains of CAR molecule, the effector cell activation, cytotoxic granules secretion leading to the tumor B cell killing. 

Recently a similar approach has been proposed for NK cells as well. Indeed, it has been planned the introduction of CARs into NK cells to boost their potent killing activity to tumor cells. These CARs recognize specific antigens on target cells and help the natural propensity of NK cells to kill tumor cells based on their expression of activating receptors [250]. Preclinical studies have shown that CAR.NK cells expressing SLAM-F7 displayed enhanced cytotoxicity, cytokine secretion, and anti-tumor activity [251,252,253]. Similar results have been observed in CAR-NK cells expressing CD19, CD20, or TRAIL [254,255,256]. Interesting results have been also obtained using NK cells genetically modified with a CD138-CAR. CD138 is a member of the syndecan family of type I transmembrane proteoglycans and is highly expressed on MM cells, where it plays an important role in their adhesion, proliferation, and angiogenesis [257,258,259,260]. These CD138-CAR NK cells displayed considerably enhanced cytotoxicity against CD138^+^ MM cell lines and primary MM cells, compared to untransduced NK cells. Additionally, this enhanced CD138-CAR NK cell-mediated killing was associated with increased secretion of Granzyme B and IFN-γ [253,259,261]. Based on these results, several clinical trials are ongoing [258,259,260]. 

**Figure 3:**
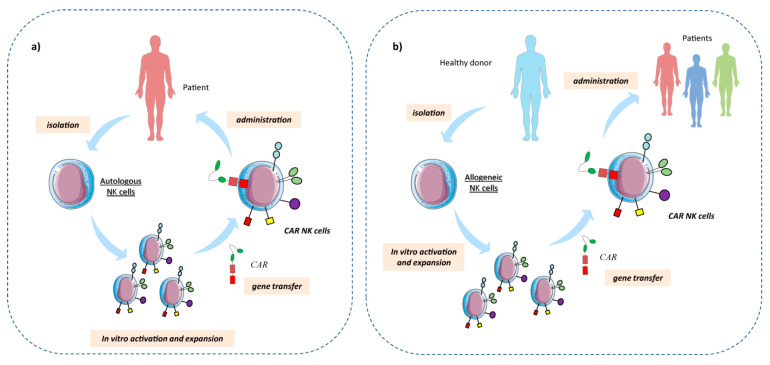
Schematic chimeric antigen receptor (CAR) NK cells therapy. (**a**)NK cells are isolated from patients (autologous), activated, expanded, and then genetically modified to express specific CARs. Therefore, autologous CAR NK cells are administered to the patient. (**b**) NK cells isolated from healthy donors (allogeneic) are activated and then genetically modified to express specific CARs and consequently expanded. Allogeneic NK cells are then administered to several patients. Allogeneic CAR NK cells can be obtained from different sources, including peripheral blood mononuclear cells (PBMC), NK cell lines, umbilical cord blood (UBC), embryonic cells (ES) or induced pluripotent stem cells (iPSC).

Based on the fact that tumor cells can increase the expression of NKG2D ligands on their surface upon stress signals but TME can simultaneously decrease the expression of NKG2D on NK cells, NKG2D-CARs have been recently developed in the attempt to further increase NK cell activation. These CAR NK cells express NKG2D combined with several co-stimulatory domains such as DNAX-activation protein (DAP)-10, 4-1BB, CD3ζ and CD28. Of note, these NK cells displayed an up-regulated expression of NKG2D and strong cytotoxicity abilities against malignant cells [247,262,263,264,265,266]. Importantly, NKG2D-CAR NK cells not only can recognize the NKG2D ligands expressed on tumor cells, but also on the other immunosuppressive cells within the TME. An interesting study reported that NK92 cells genetically modified with an extracellular domain of TGF-βRII and an intracellular domain of NKG2D were resistant to the TGF-β produced by the TME, secreted high amounts of IFN-γ, and exhibited strong killing capacity. In addition, these TGF-βRII^+^NKG2D^+^ NK cells impaired the generation of Treg populations and inhibited tumor growth [267]. Although these results have been obtained in solid tumors, this approach could also be investigated in hematological malignancies, to help infiltrated NK cells to move aside the TME’s traps. To avoid possible toxicities in patients, several groups recently started to introduce also suicide genes in engineered cells [268,269]. Interestingly, it has been recently shown that cord blood-derived NK cells expressing CD19 and the suicide gene inducible Caspase-9 (iC9) and producing IL-15 (CAR CD19/IL-15/iC9) exhibited an efficient killing of CD19^+^ tumor B cells both in vitro and in vivo [270]. To strengthen NK cell-mediated anti-tumor response and improve patient’s survival, the use of several therapeutic combinations have been evaluated. For example, CAR NK cell-based therapy in hematological malignancies has been associated with drugs as Lenalidomide [271,272] and mAbs as Elotuzumab [46,273], Nivolumab [274,275] or Pembrolizumab [276]. CAR NK cells represent a very exciting approach for cancer immunotherapy. Importantly, the advantage of the utilization of allogeneic CAR-NK cells is their “off-the-shelf” manufacturing, limited life-span, no induction of cytokine release syndrome (CRS) and do not cause graft versus host disease (GVHD) [18,210,219,247,249,250,277,278,279] (Figure 3). In addition, compared to CAR T cells, CAR NK cells will retain their ability to search and destroy targets through their natural arsenal. These features could allow allogeneic CAR NK cells to circumvent the traps found into the TME and promote their anti-tumor immune-surveillance, for an efficient treatment of hematological malignancies (Table 2). Despite all these strategies and weaponry, there are however some points that must be considered, like the optimal activating cocktail, drug combination strategies, the best source and subset population to generate ready-to-kill NK cells and the best way to enhance NK cells homing and survival at the tumor site [40,61,210,250,263,280,281,282,283,284].

### 3.2. T-Cell Redirected for Universal Cytokine-Mediated Killing (TRUCKs)

Another strategy causing great enthusiasm is represented by T-cell redirected for universal cytokine-mediated killing (TRUCK), the fourth generation of CAR-T cells developed to affect not only the tumor cells themselves but the entire TME [285,286]. Although promising results on T cells have been reported, today there are no current studies in NK cells.

## 4. Conclusions and Perspectives 

NK cells are one of the most efficient immune cell killing machines available (cit. [219]) and play a major role in tumor surveillance. Unfortunately, tumor cells and the surrounding TME always develop new tricks to escape to their killing [7,10]. Decreased recognition and cytotoxic functions of NK cells have been described in hematologic malignancies, because of diminished expression of activating receptors, cytokine secretion, and granule exocytosis [16]. In an attempt to restore NK cell-mediated anti-tumor activities, several therapeutic strategies have been developed to treat hematological malignancies. The introduction of drugs as histone deacetylases inhibitors (HDACis) [287,288,289] and IMiDs [27,287,289,290,291,292] significantly improved NK cell recognition and negatively modulated the TME-induced inhibitory functions, thus promoting the killing of tumor cells. In the past decade, other therapeutic approaches have been developed, such as checkpoint inhibitors and engineered cells. The checkpoint inhibitors approach has been validated for the treatment of most of hematological malignancies. The blockade of KIRs and the PD-1/PD-L1 axis or other promising mAbs targeting specific antigens expressed on malignant plasma cells as CD22, CD38, and SLAM-F7 have been described to relieve the exhausted status of NK cells and to restore NK cell surveillance. On other hands, engineered mAbs as BiKEs/TRiKEs remarkably arisen as promising strategies for the treatment of hematological malignancies. Engineered mAbs improve NK cell activation through CD16 and, by facilitating the formation of an immunological synapse, increase NK cell cytolytic activity against tumor cells. These BiKEs/TRiKEs obtained to such an extent promising results that are currently investigated in preclinical and clinical studies [222,223,224,225,226,227,228,229,230,231,232,233,234,277]. In hematological cancers the adoptive cell therapy based on the utilization of CAR T cells has arisen as a promising strategy. However, qualities such as natural immune-surveillance, “off-the-shelf” manufacturing, limited life-span, no CRS or GVHD induction, render CAR NK cells a therapeutic tool in the treatment of hematological malignancies [18,210,219,247,249,250,277,278,279]. A consistent indication that CAR NK cells represent a safe alternative to CAR T cells has been recently provided by Tang and colleagues, whose reported that CD33-CAR NK cells administrated to r/r AML patients have not shown significant adverse effects [293]. Nonetheless, to generate the perfect functional ready-to-use CAR NK cell, there are still many questions and reefs to pass [40,61,210,250,263,280,281,282,294].To affect tumor escape and to restore an adequate anti-tumor response is needed. Although the strategies discussed in this review have shown brilliant results, robust evidence supports the fact that these approaches should be combined altogether to maximize the chances of NK cells to exhibit a complete immune-surveillance circumventing the immunosuppressive behavior of the TME.

## Figures and Tables

**Table 1:** Summary of selected either completed or ongoing clinical trials using mAbs in hematological cancers.

Target	mAb	Drug Combination	Disease	Trial Number (NCT)	Phase
CD38	Daratumumab	Lenalidomide + Dexamethasone	MM	02076009	III
		Bortezomib + Dexamethasone	MM (CASTOR)	02136134	III
			MM (SIRIUS)	01985126	II
		Carfilzomib, Lenalidomide, Dexamethasone	newly diagnosed MM	03290950	II
		Bortezomib, Lenalidomide, Dexamethasone	untreated MM (PERSEUS)	03710603	III
		Lenalidomide, Dexamethasone	MM r/r (POLLUX)	02076009	III
		Bortezomib, Cyclophosphamide, Dexamethasone	MM r/r (LYRA)	01951819	II
		Prednisone, Bortezomib, Melphalan vs Daratumumab alone	MM r/r (ALCYONE)	02195479	III
		Lenalidomide, Dexamethasone	untreated MM (MAIA)	02252172	III
		Bortezomib, Thalidomide, Dexamethasone	untreated MM (CASSEOPEIA)	02541383	III
	Isatuximab (SAR650984)	Lenalidomide, Dexamethasone	MM	01749969	Ib
		Pomalidomide, Dexamethasone	MM r/r (ICARIA)	02990338	III
CD137	Urelumab	Nivolumab	B-cell NHL	02253992	I/II
		Elozutumab	MM	02252263	I
SLAM-F7 (CS1)	Elotuzumab		MM	03003728	II
		Nivolumab	MM r/r (Checkmate-602)	02726581	III
		Lenalidomide, Dexamethasone	MM (ELOQUENT-2)	01239797	III
KIR2DL1/2/3	IPH2102 (Lirilumab)	Nivolumab, 5-Azacytidine	Leukemia	02599649	II
		Rituximab (anti-CD20)	High-risk Untreated and r/r CLL	02481297	II
		Elotuzumab, Urelumab	MM	02252263	I
		Lenalidomide	MM	01217203	I
	IPH2101 (1-7F9)		SMM	01222286 (KIRMONO)	II
			MM	00999830 (REMYKIR)	II
			AML	01256073	I
			MM	00552396	I
		Lenalidomide	MM	01217203 (KIRIMID)	I
	MEDI4736	5-Azacytidine	Leukemia	02399917	II
	MEDI6469	Tremelimumab (anti-CTLA4) or Rituximab or MEDI4736	B-cell lymphoma, MDS	02205333	I/II
KIR3DL2	IPH4102 (Lacutamab)		Cutaneous T-cell lymphoma	02593045	I
NKG2A	IPH2201 (Monalizumab)		Hematological cancers	02921685	I
			CLL	03088059	II
			CLL	02557516	I/II
		Sym-021 (anti-PD-1), Sym-022 (anti-Lag-3)	Lymphoma	03311412	I
	MBG453	Decitabine (hypomethylating agent)	AML, high risk MDS	03066648	I
Lag-3	Sym-022		Lymphoma	03489369	I
		Sym-021 (anti-PD-1), Sym-023 (anti-Tim-3)	Lymphoma	03311412	I
	BMS-986016	Nivolumab (BMS-936558)	DLBCL r/r, HL r/r	02061761	I/II
PD-1	Pembrolizumab		cHL r/r	01953692 (Keynote-013)	Ib
			cHL r/r post ASCT	02458594 (Keynote-087)	II
		Ibrutinib	NHL r/r	02950220	I
		Brutoximab Vedotin (anti-CD30 mAb)	cHL r/r	02684292 (Keynote-024)	III
		Lenalidomide, Dexamethasone	MM	02036502 (Keynote-023)	I
		Pomalidomide, Dexamethasone	MM r/r	02576977 (Keynote-183)	III
	Nivolumab		cHL r/r (Checkmate-205)	01592370	II
		Epacadostat (anti-IDO1 mAb)	DLBCL, HL	02327078	I/II
		Lenalidomide	NHL, cHL r/r	03015896	I/II
			FL r/r (Checkmate-140)	02038946	II
		Lenalidomide, Rituximab	DLBCL	03558750	I
			DLBCL r/r (CheckMate-139)	02038933	II
		Cyclophosphamide, Prednisone, Doxorubicin Hydrochloride	DLCBL	03704714	I/II
		Urelumab (anti-CD137 mAb)	NHL	02253992	I/II
		Varlilumab (CDX-1127) (anti-CD27 mAb)	DLCBL	03038672	II
			HL r/r (ANIMATE)	03337919	II
		Lenalidomide, Dexamethasone	high risk SMM	02903381	II
		Lenalidomide	MM r/r	03333746	II
		Daratumumab with or without Cyclophosphamide	MM r/r	03184194	II
		Daratumumab or Pomalidomide and Dexamethasone	Hematological cancers	01592370	I
		Ipilimumab (anti-CTLA4)	high risk MM	02681302	I/II
		Elotuzumab with or without Pomalidomide and Dexamethasone	MM r/r	03227432	II
		Elotuzumab, Pomalidomide, Dexamethasone	MM r/r	02726581	III
			AML	02275533	II
		Dasatinib (tyrosine kinase receptor inhibitor)	CML	02011945	I
			HL	02181738	II
		Rituximab, Gemcitabine, Bendamustine (alkylating agent)	DLBCL r/r	03259529	I/II
	Pidilizumab	Rituximab	FL r/r	00904722	II
		Lenalidomide	MM	02077959	I/II
PD-L1	Atezolizumab	Obinutuzumab (anti-CD20)	DLBCL, FL r/r	02220842	I
		Obinutuzumab, Polatuzumab vedotin (anti-CD79b mAb)	DLBCL, FL r/r	02729896	I
		Obinutuzumab, Lenalidomide	FL r/r	02631577	I
		Obinutuzumab, Ibrutinib	untreated, high risk or r/r CLL	02846623	II
		Guadecitabine (hypomethylating agent)	AML, MDS, CML r/r	02935361	I/II
		Daratumumab vs Daratumumab, IMiDs	MM	02431208	I
	Avelumab		cHL r/r	02603419	Ib
		Itolizumab (anti-CD6) vs Itolizumab, 5-Azacytidine vs Bendamustine, Rituximab	DLBCL r/r	02951156	Ib/II
		5-Azacytidine	AML r/r	02953561	I
	Cetrelimab (JNJ-63723283)	Daratumumab	MM r/r	03357952	II/III
	Durvalumab	Daratumumab	MM r/r	03000452	II
		Lenalidomide, Dexamethasone	newly diagnosed MM	02685826	I
		Pomalidomide, Dexamethasone	MM r/r	02616640	I
		Rituximab, Lenalidomide with or without Ibrutinib	NHL, CLL	02733042	I/II

Abbreviations used: ALL, Acute Lymphocytic Leukemia; AML, Acute Myeloid Leukemia; CLL, Chronic Lymphocytic Leukemia; CML, Chronic Myeloid Leukemia; MM, Multiple Myeloma; MDS, Myelodysplastic Syndromes; NHL, Non-Hodgkin Lymphoma; FL, Follicular Lymphoma; DLBCL, Diffuse Large B-Cell Lymphoma; SMM, Smoldering MM; r/r, refractory/relapsed; IMiDs, immunomodulatory drugs.

**Table 2:** Summary of current clinical trials using CAR NK cells in hematological cancers.

Sources of NK Cells	Disease	Receptor Target	Trial Number (NCT)	Phase
NK92	Lymphoma and Leukemia	CD7	02742727	I/II
	Lymphoma and Leukemia	CD19	02892695	I/II
	AML r/r	CD33	02944162	I/II
	MM r/r	BCMA	03940833	I/II
	B-cell lymphoma r/r	CD19	03690310	I
CB-derived NK cells	ALL, CLL and NHL r/r	CD19	03056339	I/II
	B-cell lymphoma	CD19	03579927	I/II
unknown	B-cell lymphoma r/r	CD19+CD22	03824964	I
PB NK cells	ALL	CD19	01974479	I
	ALL	CD19	00995137	I
unknown	B-cell lymphoma r/r	CD22	03692767	I
iPSC-derived NK cells	B-cell lymphoma r/r	CD19	03824951	I

Abbreviations used: ALL, acute lymphocytic leukemia; AML, acute myeloid leukemia; CLL, chronic lymphocytic leukemia; MM, multiple myeloma; NHL, non-Hodgkin lymphoma; r/r, refractory/relapsed; CB, cord blood; iPSC, induced pluripotent stem cells; PB, peripheral blood; BCMA, B cell maturation antigen.

## Abbreviations

ALL: Acute Lymphocytic Leukemia; AML, Acute Myeloid Leukemia; CLL, Chronic Lymphocytic Leukemia; CML, Chronic Myeloid Leukemia; MM, Multiple Myeloma; MDS, Myelodysplastic Syndromes; NHL, Non-Hodgkin Lymphoma; FL, Follicular Lymphoma; DLBCL, Diffuse Large B-Cell Lymphoma; SMM, Smoldering MM; r/r, refractory/relapsed; IMiDs, immunomodulatory drugs
